# Androgen insensitivity syndrome: A case report

**DOI:** 10.1097/MD.0000000000050953

**Published:** 2026-10-02

**Authors:** Yong Zhang, Houmei Wang, Ziwen Xiao

**Affiliations:** a School of Clinical Medicine, The Guizhou Medical University, Guiyang, China; b Department of Gynecology, The First People’s Hospital of Guiyang, Guiyang, China; c Department of Obstetrics and Gynecology, The Affiliated Hospital of Guizhou Medical University, Guiyang, China.

**Keywords:** 17*β*-Hydroxysteroid dehydrogenase deficiency, 46, XY disorder of sex development, 5*ɑ*-reductase deficiency, Androgen insensitivity syndrome, Missed diagnosis

## Abstract

**Rationale:**

46, XY disorder of sex development (46, XY DSD) is relatively rare and frequently missed in clinical practice, necessitating early standardized diagnosis and treatment. Androgen insensitivity syndrome (AIS), a major subtype, should be considered in phenotypic females with suggestive clinical clues.

**Patient concerns:**

A 29-year-old phenotypic female presented with primary amenorrhea and intermittent abdominal pain. Examination revealed normal breast development, sparse pubic hair, and female external genitalia. Pelvic ultrasonography and CT showed an absent uterus and ovaries, with bilateral inguinal masses.

**Diagnoses:**

The case met the 3 diagnostic criteria for 46, XY DSD: 46,XY karyotype, testicular tissue, and feminized external genitalia. A diagnosis clinically consistent with complete AIS was further supported by the characteristic phenotype – female appearance, normal breast development, sparse pubic hair, and absent Müllerian structures. Other etiologies of undervirilization, including 5*α*-reductase deficiency and 17*β*-hydroxysteroid dehydrogenase deficiency, were considered. Genetic confirmation was unavailable, limiting subtype certainty.

**Interventions:**

Following counseling, the patient underwent laparoscopic bilateral orchiectomy as prophylactic gonadectomy to reduce malignancy risk, together with inguinal hernia repair, followed by postoperative hormone replacement therapy. Postoperative histopathology confirmed testicular tissue with Leydig cell hyperplasia, absent spermatogenic cells, and no malignancy.

**Outcomes:**

Abdominal pain resolved postoperatively, with improved psychological well-being and adaptation to the diagnosis under ongoing endocrine and psychological support.

**Lessons:**

46, XY DSD is underdiagnosed due to phenotypic variability, reliance on specialized tests, and social biases. AIS should be considered in the differential diagnosis of phenotypic females presenting with primary amenorrhea, developed secondary sexual characteristics, and an absent uterus on ultrasound. However, definitive subtyping requires AR gene sequencing, which was not available in this case. Early diagnosis requires careful assessment of newborn external genitalia, with specialist evaluation prompted by any atypical findings, and hormonal and genetic testing in adolescents with abnormal development. Multidisciplinary care – involving gynecologists, endocrinologists, surgeons, geneticists, and mental-health professionals – is essential for comprehensive management, including psychological support, hormone therapy, and timely prophylactic gonadectomy. Establishing specialized multidisciplinary centers for DSD is also recommended.

## 1. Introduction

Human sex differentiation is a complex biological process primarily determined by the karyotype of sex chromosomes, the structure of gonads, the morphology of external genitalia, and the presence of secondary sexual characteristics. However, some individuals exhibit a coexistence of sexual characteristics, a condition clinically referred to as intersex.^[1]^ In 1953, Morris provided a systematic description of the clinical characteristics of androgen insensitivity syndrome (AIS), establishing a foundational understanding of this condition.^[2]^ Patients with 46, XY DSD exhibit the following characteristics: a 46,XY chromosome karyotype, differentiated testicular tissue, and abnormal development of the external genitalia resulting from disorders in androgen synthesis or action.^[3]^ These patients may present with varying degrees of feminine manifestations, and during adolescence, they may develop either incomplete or complete female secondary sexual characteristics.^[4,5]^ Epidemiological data indicate that the incidence of this condition among male newborns ranges from approximately 1 in 20,000 to 1 in 64,000.^[6]^ The occurrence of 46, XY DSD may be associated with mutations (specifically p.P913R, p.D732N, p.N848K, fs879X, and p.R608K) or deletion (11P3) in androgen receptor genes, deletions of the steroid 5*α*-reductase 2 gene (SRD5A2), and homozygous missense mutations (Ala593Pro) in the luteinizing hormone receptor gene. Additionally, it is linked to the missense mutation (R80W) of the 17*β*-hydroxysteroid dehydrogenase type 3 gene; these findings suggest a certain genetic predisposition to this condition.^[7–11]^ The pathogenesis primarily involves abnormal androgen biosynthesis pathways or defective responses of target tissues to androgens.^[5]^

In this report, we present a rare adult case of 46, XY DSD that underwent comprehensive clinical evaluation, including physical examination, karyotyping, hormonal assays, imaging, and histopathological analysis. This report not only delineates the atypical presentation in adulthood but, more importantly, underscores the diagnostic challenges and instructive takeaways derived from delayed recognition, with the objective of sharpening clinicians’ vigilance and clinical reasoning in analogous scenarios. Moreover, the clinical significance of early detection in 46, XY DSD cannot be overstated. Timely diagnosis enables the implementation of multidisciplinary care encompassing psychological support for gender identity and psychosocial adjustment, appropriate hormone replacement therapy to maintain endocrine homeostasis, and planned gonadectomy at an optimal time to mitigate the risk of gonadal malignancy. Emphasizing these interventions is essential for improving long-term outcomes and underscores the educational value of this case report for clinicians, particularly in resource-limited settings where molecular confirmation may not be readily available.

## 2. Case presentation

The patient is a 29-year-old female who presented with a female vulvar appearance at birth. During adolescence, she experienced breast development; however, menstruation did not occur. The patient presented to a specific hospital 10 years ago due to amenorrhea. A gynecological ultrasound examination revealed “congenital absence of the uterus with the presence of ovaries.” There were no reports of periodic abdominal pain or distension. Upon reaching adulthood, she indicated that there were no impediments to her sexual life. Consequently, regular follow-ups were deemed unnecessary. One month prior, she sought medical attention at another hospital due to complaints of abdominal pain. An abdominal CT scan revealed the absence of both the uterus and ovaries. Of note, an earlier ultrasound at the county hospital had reportedly suggested “ovarian-like structures”; however, this was attributed to misinterpretation by the local sonographer, who likely mistook the inguinal testes for ovarian tissue. The differential diagnosis of other causes of undervirilization, including 5*α*-reductase deficiency and 17*β*-hydroxysteroid dehydrogenase deficiency, was considered; however, the clinical and biochemical profile was most consistent with AIS. Written consent was obtained from the patient for the publication of this case report and the accompanying images. The karyotype analysis indicated a chromosomal pattern of 46, XY (Fig. 1). Subsequently, the patient was admitted to our hospital on December 9, 2023, for further diagnosis and treatment, where she was diagnosed with 46, XY DSD upon admission.

**Figure 1.**
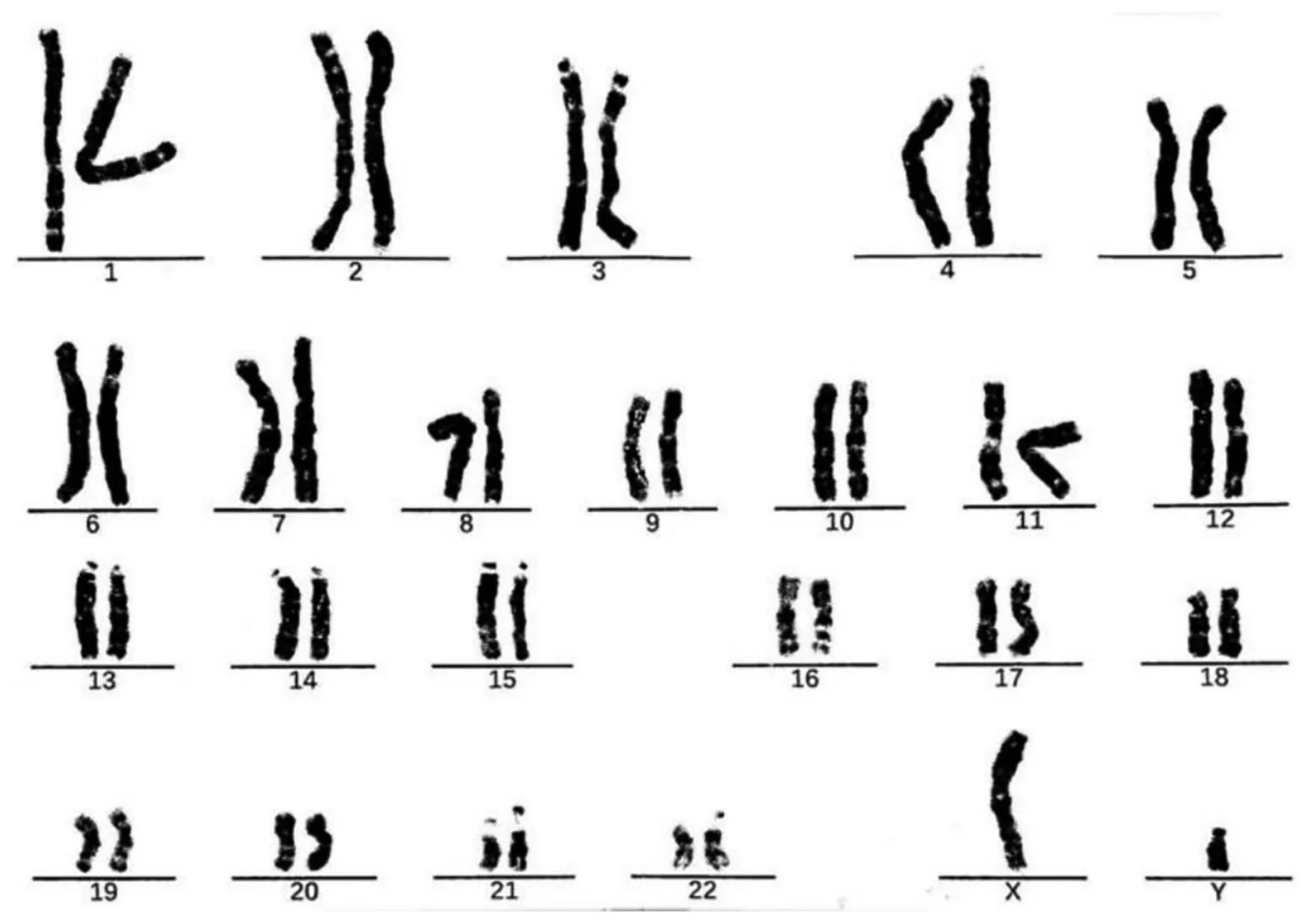
Karyotype analysis.

Upon admission, the physical examination revealed a weight of 48 kg and a height of 162 cm, indicating a relatively thin build. Laryngeal prominence was visible, but the voice remained unaffected and did not exhibit hoarseness. Breast development was assessed at Tanner stage IV. The vulva appeared immature and had sparse pubic hair. The vagina was unobstructed but measured only 6 cm in length and had a blind tip. The pelvic cavity was empty, with no palpable uterus or appendages. Upon standing and coughing, masses approximately 3 × 2 × 1 cm were palpated in both groin areas. B-ultrasound and magnetic resonance imaging of the pelvic cavity suggested the presence of cryptorchidism (Figs. 2 and 3). These masses were solid, had clear boundaries, exhibited mild tenderness, and demonstrated good mobility.

**Figure 2.**
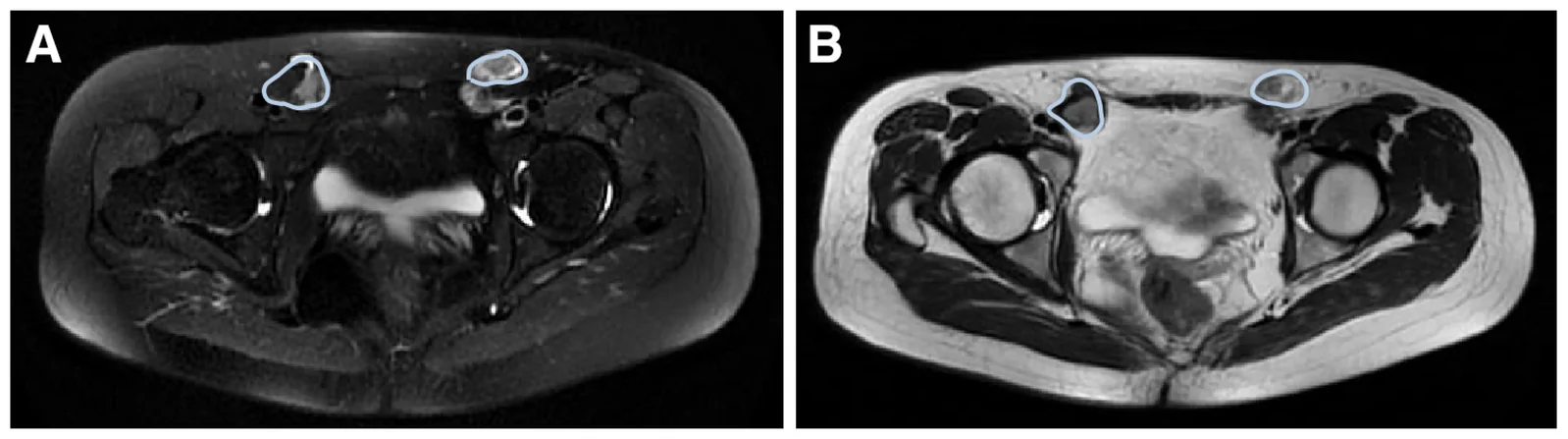
Magnetic resonance imaging of the pelvic cavity: (A) T1-WI image; (B) T2-WI image. The part framed by the light blue lines is cryptorchidism.

**Figure 3.**
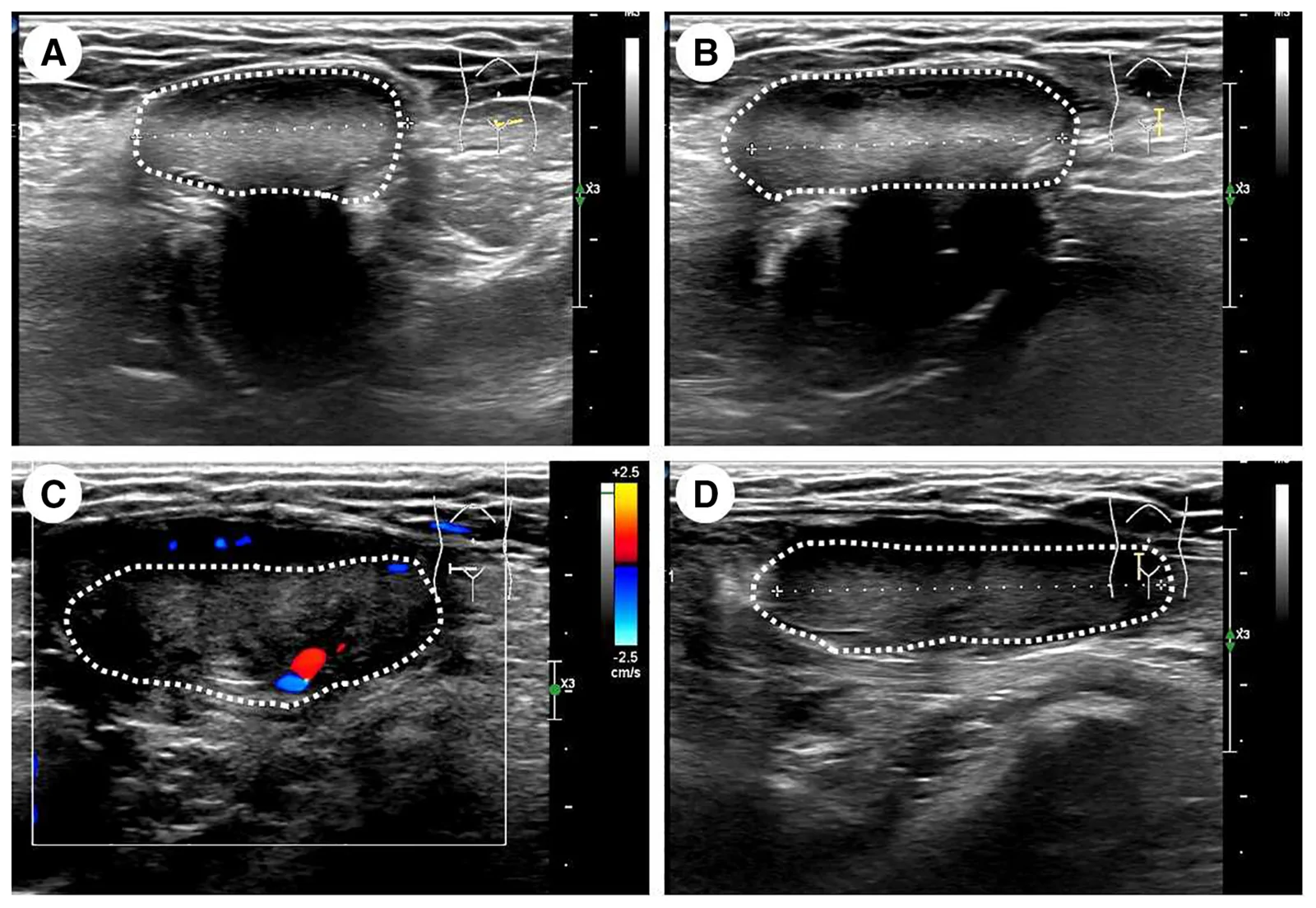
Preoperative color Doppler ultrasound localization and morphological assessment of undescended testes: (A) Transverse view of the left undescended testis; (B) Longitudinal view of the left undescended testis; (C) Transverse view of the right undescended testis; and (D) Longitudinal view of the right undescended testis. The white dotted line marks undescended testes.

Auxiliary examinations revealed that 6 sex hormone items – follicle-stimulating hormone (FSH) elevated, luteinizing hormone (LH) elevated, estrogen decreased, androgen decreased, progesterone elevated, and prolactin elevated – along with routine blood, urine, and stool tests, liver and kidney function assessments, and electrolyte levels, showed no abnormalities. The elevated prolactin was mild and asymptomatic; it was attributed to stress-related causes, and no further investigations were performed, as the patient had no clinical features suggestive of a pituitary disorder. Additionally, no significant anomalies were detected in the anteroposterior and lateral positions of the chest. An adrenal ultrasound and color Doppler ultrasound of the liver, gallbladder, pancreas, spleen, bilateral kidneys, ureters, and bladder also indicated no notable abnormalities.

Following comprehensive discussions with the patient and their family regarding the condition and the indications for prophylactic bilateral gonadectomy to reduce the risk of gonadal malignancy, written informed consent was obtained. After ruling out any contraindications for surgery, laparoscopic exploration, bilateral orchiectomy, and inguinal hernia repair were conducted under general anesthesia on December 11, 2023.

The postoperative pathological examination results indicated the following: In the pathological section of the right undescended testis, no spermatogenic cells of any grade were identified in the seminiferous tubules of the testicular tissue, nor was any sperm detected in the lumen. Furthermore, there was notable hyperplasia of interstitial cells and fibrous tissue. Focal nodular hyperplasia of immature testicular tissue was observed, which aligns with the pathological changes associated with undescended testis. In the pathological section of the left undescended testis, similar findings were noted, with no spermatogenic cells of any grade present in the seminiferous tubules, absence of sperm in the lumen, and significant proliferation of interstitial cells and fibrous tissue, consistent with the changes associated with undescended testis (Fig. 4).

**Figure 4.**
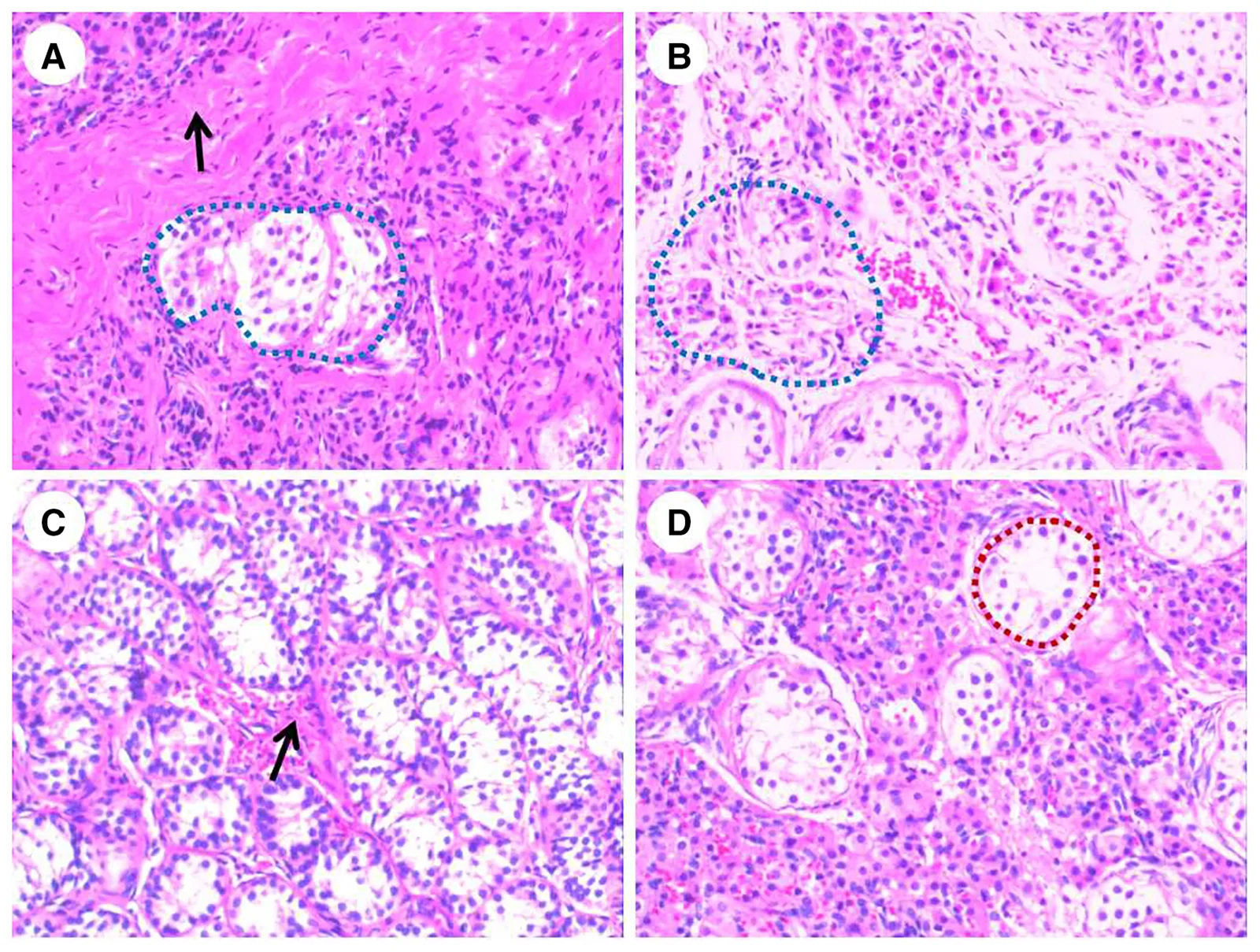
HE staining of cryptorchidism: (A) Left cryptorchidism (100×) and (B) 200×; (C) Right cryptorchidism (100×) and (D) 200×. The black arrow indicates fibrous tissue, the blue dotted line represents proliferative nodules, and the red dotted line delineates seminiferous tubules.

Following the initiation of hormone replacement therapy with a daily dose of 0.5 mg of estradiol valerate, regular gynecological outpatient follow-ups were scheduled. In the most recent follow-up, the patient reported no recurrence of abdominal pain, and she described improved psychological well-being and adaptation to the diagnosis following treatment.

## 3. Discussion

46, XY DSD (historically termed male pseudohermaphroditism) is defined by a 46,XY karyotype, the presence of testicular tissue, and feminized or ambiguous external genitalia.^[12]^ The etiological mechanisms underlying this condition are multifactorial and can be classified into 3 categories^[13]^ – abnormal testicular development (e.g., SRY/SOX9 gene defects); androgen dysfunction (mutations in the androgen receptor gene) or dyssynthesis (deficiencies in 5*α*-reductase, 17*β*-hydroxysteroid dehydrogenase, or 17*α*-hydroxylase); and persistent Müllerian duct syndrome (due to anti-Müllerian hormone pathway abnormalities). This classification provides a crucial framework for clinical diagnosis.

In this report, the clinical phenotype of normal breast development, sparse pubic and axillary hair, absent uterus, and bilateral inguinal testes is consistent with complete AIS. The hormonal findings, together with normal blood pressure and normal electrolyte levels, do not support 17*α*‑hydroxylase deficiency, which would typically present with mineralocorticoid excess and hypokalemia. The elevated progesterone observed was therefore considered incidental. However, the lack of molecular confirmation inevitably reduces diagnostic certainty. The mildly elevated prolactin observed in this case was asymptomatic and considered stress-related, as the patient had no galactorrhea, visual disturbances, or other signs of hyperprolactinemia, and no prolactin-elevating medications were being used. Given the mild elevation and lack of clinical indications, no pituitary imaging was pursued. Thus, this finding was not considered clinically significant to the primary diagnosis.

This diagnostic pattern should prompt early clinical recognition of complete AIS. While complete AIS is typically diagnosed during adolescence following investigation of primary amenorrhea, our patient presented at 29 years of age, illustrating the diagnostic delays that can occur when the condition remains unrecognized in childhood. In comparison with previously reported cases, our patient shares the common features of complete AIS, including female phenotype, 46,XY karyotype, normal breast development, and absent Müllerian structures, though her age at diagnosis is substantially later than the typical adolescent presentation. This delayed diagnosis underscores the importance of heightened clinical suspicion in adult patients presenting with primary amenorrhea or infertility, and reinforces the educational value of this report for clinicians across different specialties. Definitive confirmation of complete AIS would require identification of an AR gene mutation, which was not available in this case; the diagnosis therefore remains clinical, albeit strongly supported by the phenotypic and hormonal profile.

The diagnosis of 46, XY DSD necessitates a multidisciplinary collaboration model that systematically integrates clinical phenotypes, endocrine evaluations, imaging features, and molecular genetic test results. According to the “Clinical Practice Guidelines for Sexual Developmental Disorders (DSD)” (2016),^[6]^ the standardized assessment of neonatal external genital ambiguity should encompass –Chromosomal karyotype analysis; Simultaneous evaluation of hormone profiles, including serum 17-hydroxyprogesterone, testosterone, dihydrotestosterone, and AMH, which are crucial for differentiating congenital adrenal hyperplasia (CAH) from other DSD subtypes. In newborns with atypical or ambiguous genitalia, or other findings suggestive of DSD, prompt evaluation by a specialist multidisciplinary team is recommended. Imaging assessments, such as pelvic ultrasound and MRI, can clarify the location of the gonads and the developmental status of the Müllerian ducts. Additionally, molecular genetic testing, including whole exome sequencing or targeted gene panels, can significantly enhance the detection rate of pathogenic genes, thereby providing a foundation for precise classification.

Special attention should be paid to avoiding misjudgment of excessive gendering in clinical diagnoses, particularly in the subtype differentiation of suspected AIS, which is crucial. Patients with complete AIS (CAIS) typically present with female external genitalia, whereas those with partial AIS (PAIS) may exhibit atypical features such as clitoral hypertrophy or partial fusion of the labia and scrotal folds due to some retained androgen sensitivity. It is important to note that mutations in the SRD5A2 gene (5*α*-reductase deficiency) or the LHCGR gene (luteinizing hormone/chorionic gonadotropin receptor) can result in phenotypes resembling PAIS. These conditions must be differentiated through molecular genetic testing due to significant differences in their pathological mechanisms and clinical management principles.^[14]^

Management of 46, XY DSD requires a comprehensive, individualized approach. The timing of prophylactic gonadectomy should be carefully considered, balancing the age-related risk of gonadal malignancy against the potential benefits of preserving spontaneous pubertal development. In patients diagnosed before puberty, gonadectomy may be deferred to allow endogenous pubertal development, whereas in postpubertal patients such as ours, the decision is less complex. Following gonadectomy, long-term hormone replacement therapy is essential to maintain endocrine homeostasis, preserve bone mineral density, and prevent osteoporosis, cardiovascular disease, and other complications of hypogonadism. Beyond endocrine management, comprehensive care should address bone health, sexual health (including vaginal anatomy and potential need for vaginal dilation or surgical correction), fertility limitations (absolute due to the absence of a uterus), and psychological well-being. Shared decision-making, with the patient fully informed and involved in all treatment choices, is paramount to achieving optimal outcomes.

The treatment of 46, XY DSD should adhere to the principle of “patient-centered and multidisciplinary collaboration.” According to international consensus,^[1]^ the treatment goals include – supporting the patient’s affirmed gender identity and facilitating psychological adaptation to the diagnosis through individualized psychosocial support; preventing gonadal tumors; improving reproductive potential (if feasible); and providing long-term endocrine support. In this case, the patient’s social gender is identified as female, based on her own and her family’s wishes and the feminization tendencies of the external genitalia. However, it is important to note that the gender identity of patients with PAIS may fluctuate. Studies indicate that approximately 20% of PAIS patients experience gender anxiety after adolescence. Consequently, the European Society of Paediatric Endocrinology recommends that only necessary surgical interventions, such as cryptorchidopexy, be performed during infancy and early childhood, while delaying any external genitalia surgery until the patient is capable of making autonomous decisions.

Although patients with PAIS typically exhibit extremely low fertility, it is possible to achieve offspring through assisted reproductive technologies, such as testicular sperm extraction. This patient necessitates long-term estrogen replacement therapy (ERT) following gonadectomy to preserve bone mineral density and promote the development of secondary sexual characteristics.

The patient received thorough counseling regarding the diagnosis, the rationale for prophylactic gonadectomy, surgical risks and benefits, and the necessity of lifelong hormone replacement therapy and regular long-term follow-up. This comprehensive counseling process is essential to ensure informed decision-making and to optimize the patient’s understanding of and adherence to the management plan.

The risk of malignant transformation associated with cryptorchidism in patients with 46, XY DSD exhibits significant heterogeneity. Current evidence indicates that the incidence of gonadal malignancy in patients with partial androgen insensitivity syndrome (PAIS) ranges from 3% to 5%, whereas in those with complete androgen insensitivity syndrome (CAIS), it is reported to be between 0.8% and 2%.^[15]^ Notably, no malignant evidence was detected in the histopathological examination of this case following laparoscopic bilateral orchiectomy. According to the latest clinical guideline recommendations, patients with preserved gonads are advised to undergo regular ultrasound monitoring and serum tumor marker detection (AFP, *β*-hCG). For high-risk patients with identifiable genetic susceptibility, preventive adenectomy is recommended post-puberty.

The lifelong follow-up of patients with DSD must encompass multiple dimensions, including endocrinology, urology and reproduction, psychology, and social adaptation. Research conducted by Ahmed et al indicates that the establishment of a specialized outpatient clinic for DSD can significantly enhance the quality of life for these patients.^[16]^ Furthermore, genetic counseling plays a crucial role in this context. The AR gene mutation follows an X-linked recessive inheritance pattern, which implies that male offspring of female carriers face a 50% risk of developing the condition. To mitigate the recurrence risk, prenatal diagnosis or preimplantation genetic testing (PGT) is essential.

The diagnosis of this case was delayed until the age of 29 due to a combination of multiple factors. Firstly, as a rare disease, the abnormal development of the vulva at birth may not be readily apparent, leading to its oversight during routine neonatal examinations. Secondly, the patient has identified as female since childhood and exhibited no significant developmental abnormalities prior to puberty. Family and social psychological factors contributed to a lack of motivation for seeking medical treatment. Additionally, routine pediatric monitoring typically concentrates on common diseases and indicators of growth and development. In the absence of obvious abnormalities in the external genitalia, comprehensive examinations, such as chromosome tests, are rarely performed. Ultimately, the clear postadolescent clinical manifestation of “primary amenorrhea” served as the pivotal event that prompted this diagnosis. This case underscores the importance of maintaining clinical awareness of suggestive features regarding external genitalia in newborns and highlights the diagnostic challenges that can arise when such features are subtle or unrecognized.

It is essential to acknowledge the inherent limitations of this case series. Firstly, the incidence of 46, XY DSD is extremely low, making it challenging to represent the full spectrum of the disease with a single case report. Due to the patient’s personal reasons, the gene sequencing could not be completed. Consequently, this case represents a clinical diagnosis rather than a genetic confirmation. The clinical manifestations vary significantly depending on the underlying causes, and conclusions drawn from individual cases may not be applicable to all subtypes. Secondly, there is a notable lack of long-term follow-up data, particularly concerning indicators such as postoperative quality of life, stability of gender identity, and the risk of gonadal malignancy. Finally, the diagnosis in this case was based on karyotype and pathological examination; however, genetic testing was not performed, which has resulted in an unclear molecular mechanism and has implications for the genetic counseling of the family. Future research will focus on developing a comprehensive “full-cycle precise management”system that encompasses genetic diagnosis and social integration. By integrating multi-omics technologies, artificial intelligence-assisted decision-making, and interdisciplinary lifelong health management, the primary objectives of early intervention, personalized treatment, and enhancement of social adaptability will be accomplished.

This case report serves to illustrate the clinical presentation and diagnostic considerations in a patient with suspected complete AIS diagnosed in adulthood. It highlights the value of a systematic clinical approach in patients presenting with primary amenorrhea and suggestive phenotypic features, particularly in settings where molecular confirmation may not be readily available.

## 4. Conclusion

46, XY DSD encompasses a heterogeneous group of conditions, including disorders of gonadal development, androgen synthesis or action, and other pathways. It has a low incidence and is frequently missed in clinical practice. Although molecular testing is unavailable in some settings, the diagnosis can be preliminarily established through systematic clinical and hormonal evaluation. Clinicians should consider AIS in the differential diagnosis of a phenotypic female presenting with primary amenorrhea, normal breast development, sparse pubic and axillary hair, absent uterus, and intra-abdominal or inguinal gonads. Multidisciplinary care involving gynecologists, endocrinologists, surgeons, geneticists, and mental-health professionals is essential. For newborns with external genital abnormalities, early vigilance is critical. Comprehensive management should include psychological support, genetic counseling, individualized surgery, hormone replacement, and long-term monitoring of bone, sexual, reproductive, and general health. Treatment should be individualized, considering gender identity, tumor risk, and psychosocial well-being, with emphasis on multidisciplinary collaboration and lifelong follow-up. Molecular confirmation via AR gene sequencing remains the gold standard for definitive diagnosis.

## Acknowledgments

This study was supported by the Guizhou Provincial Science and Technology Plan Project (Qian Ke He Ji Chu-ZK[2022] General 436) and Science and Technology Fund Project of the Health Commission of Guizhou Province (GZWKJ2026-363).

## Author contributions

**Conceptualization:** Yong Zhang, Ziwen Xiao.

**Data curation:** Yong Zhang, Houmei Wang.

**Formal analysis:** Houmei Wang.

**Methodology:** Yong Zhang.

**Writing – review & editing:** Yong Zhang, Ziwen Xiao.

**Funding acquisition:** Ziwen Xiao.

**Project administration:** Ziwen Xiao.

**Supervision:** Ziwen Xiao.
